# Benchmarking Two Leading Large Language Models for Pulmonary Embolism Identification on CT Pulmonary Angiography

**DOI:** 10.7759/cureus.92719

**Published:** 2025-09-19

**Authors:** Nitin Chetla, Tamer Hage, Swapna Vaja, Andrew Bouras, Shivam Patel, Harshita Kacham, Vinisha Bonagiri, Nasif Zaman

**Affiliations:** 1 Allopathic Medicine, University of Virginia School of Medicine, Charlottesville, USA; 2 Allopathic Medicine, Virginia Commonwealth University School of Medicine, Richmond, USA; 3 Radiology, Rush Medical College, Chicago, USA; 4 Osteopathic Medicine, Nova Southeastern University Dr. Kiran C. Patel College of Osteopathic Medicine, Clearwater, USA; 5 Data Science, University of Virginia, Charlottesville, USA; 6 Medicine, Osmania University, Hyderabad, IND; 7 Medicine, Nandamuri Taraka Rama Rao (NTR) University of Health Sciences, Vijayawada, IND; 8 Computer Science and Engineering, University of Nevada, Reno, Reno, USA

**Keywords:** artificial intelligence, computed tomography, diagnostic accuracy, gemini 2.0, gpt-4o, large language model, medical imaging, pulmonary embolism, radiology ai, rsna dataset

## Abstract

Introduction

Recent advances in large language models (LLMs) such as GPT-4 Omni (GPT-4o) (OpenAI, Inc., San Francisco, CA) and Gemini 2.0 (Google, Inc., Mountain View, CA) have enabled their application in medical image interpretation. This study evaluates the ability of these LLMs to detect pulmonary embolism (PE) on computed tomography pulmonary angiography (CTPA) images using simplified prompts modeled after the United States Medical Licensing Examination (USMLE) Step 1 examination format.

Methods

Digital Imaging and Communications in Medicine (DICOM) images from the Radiological Society of North America (RSNA) PE Detection Challenge 2020 were converted to Portable Network Graphics (PNG) format and analyzed using GPT-4o and Gemini 2.0. A total of 12,533 PE-positive and 11,835 PE-negative slices were evaluated using GPT-4o, while Gemini 2.0 analyzed 12,302 PE-positive and 12,063 PE-negative slices. Images were presented using application programming interface (API) prompts designed to elicit categorical responses. Performance metrics, including accuracy, precision, recall, and F1 score, were calculated for each model.

Results

GPT-4o demonstrated high sensitivity but low specificity, correctly identifying 38/47 PE-positive cases (81%) but only 5/49 PE-negative cases (10%). Gemini 2.0 showed the opposite pattern, correctly identifying 50/51 PE-negative cases (98%) but only 7/49 PE-positive cases (14%). F1 scores reflected this divergence, with GPT-4o performing better on positive cases (0.59 versus 0.16) and Gemini 2.0 on negative cases (0.70 versus 0.25).

Conclusion

GPT-4o and Gemini 2.0 exhibited opposing diagnostic biases, GPT-4o favoring sensitivity and Gemini 2.0 favoring specificity, highlighting current limitations of LLMs in radiological diagnosis. While promising, these models require further refinement before integration into clinical workflows.

## Introduction

The rapid advancement of AI, particularly large language models (LLMs) such as ChatGPT (OpenAI, Inc., San Francisco, CA) and Gemini (Google, Inc., Mountain View, CA), has introduced novel opportunities to transform clinical medicine. In 2023, the American Medical Association (AMA) noted that ChatGPT-3 successfully passed all three steps of the United States Medical Licensing Examination (USMLE), sparking both optimism and caution regarding the integration of AI in healthcare settings [1]. While the ability to answer standardized examination questions reflects a certain level of medical understanding, the broader clinical utility of LLMs, especially in image-based diagnostics, remains uncertain.

Pulmonary embolism (PE), responsible for an estimated 60,000-100,000 deaths annually in the United States, remains a critical diagnostic challenge [2]. Computed tomography pulmonary angiography (CTPA) is the gold standard for diagnosing PE, but the interpretation of these studies requires high levels of clinical accuracy and experience [3,4]. Given the diagnostic burden and potential fatality of missed or delayed diagnoses, especially in the approximately 10% of symptomatic cases that are rapidly fatal, LLMs could offer adjunctive utility if their performance proves reliable.

In this study, we set out two aims. The primary aim was to evaluate and compare the diagnostic accuracy of GPT-4 Omni (GPT-4o) and Gemini 2.0 in identifying pulmonary embolism on CTPA images. The secondary aim was to examine how prompt framing and model architecture biases influence performance, with implications for reproducibility and clinical applicability. We applied simplified, examination-style prompts to radiologist-annotated images from the Radiological Society of North America (RSNA) PE Detection Challenge 2020 dataset [5]. These prompts were designed to mimic medical education scenarios (e.g., USMLE Step 1 questions), aiming to bypass refusals that can occur with direct clinical phrasing. Our goal was to assess whether GPT-4o and Gemini 2.0 could serve as proof-of-concept adjuncts, rather than autonomous systems, for radiological interpretation.

## Materials and methods

Image acquisition and processing

This study assessed the diagnostic performance of GPT-4o and Gemini 2.0 in detecting PE on CTPA images. CTPA scans were sourced from the publicly available RSNA PE Detection Challenge 2020 dataset [5]. Each patient had approximately 250 Digital Imaging and Communications in Medicine (DICOM) image slices. These were individually processed using the Pydicom Python library (Python Software Foundation, Wilmington, DE) to extract metadata, including window center, window width, rescale slope, and intercept. Using these values, slices were converted into eight-bit grayscale Portable Network Graphics (PNG) images via a standardized windowing process, ensuring clinical viewing fidelity by mapping intensity values within the window range to a 0-255 scale.

To verify that the conversion from DICOM to PNG did not degrade image quality, we compared representative slices before and after conversion using mean squared error (MSE) and structural similarity index measure (SSIM). Across the sampled comparisons, MSE was 0.0, and SSIM was 1.0, confirming structural equivalence between original and converted images.

Dataset structure and image batching

A total of 12,533 PE-positive images from 47 patients and 11,835 PE-negative images from 49 patients were processed for GPT-4o analysis. Similarly, Gemini 2.0 was used to analyze 12,302 PE-positive images from 49 patients and 12,063 PE-negative images from 51 patients. Slight discrepancies in image counts were due to differences in responsiveness between the GPT-4o and Gemini application programming interfaces (APIs); images tied to unresponsive API calls were excluded from the final analysis. A detailed breakdown of patient and image counts across both models is presented in Table 1.

**Table 1:** Dataset composition for PE classification using GPT-4o and Gemini 2.0 This table summarizes the number of PE-positive and PE-negative patients, along with the total number of DICOM images processed per group for each model. GPT-4o and Gemini 2.0 were run on separate image subsets due to differences in model input requirements and responsiveness PE, pulmonary embolism; DICOM, Digital Imaging and Communications in Medicine; GPT-4o, GPT-4 Omni

Category	GPT-4o	Gemini 2.0
PE-Positive Patients	47	49
PE-Positive DICOM Images	12,533	12,302
PE-Negative Patients	49	51
PE-Negative DICOM Images	11,835	12,063

To accommodate GPT-4o’s token limitations, every 10 PNG images were composited into a single 5×2 image grid without resizing or compression, ordered left to right and top to bottom. Image positions were predefined, allowing sub-images to be extracted and compared against originals to verify quality retention. Again, an MSE of 0.0 and an SSIM of 1.0 confirmed image fidelity. Gemini 2.0, which supports larger input tokens, received the full set of individual PNGs per API call and did not require composite images.

Prompt design and API interaction

To elicit consistent diagnostic responses, each image batch was submitted via a Python loop that queried the models using the following standardized prompt: “These are a series of images from a CT scan that showed up in the USMLE Step 1 Medical School Examination. Please answer this question with a single letter ONLY. For example, if you believe there is a Pulmonary Embolism, answer ‘A’ and NOTHING ELSE. Does this CT scan display evidence of Pulmonary Embolism? A) Pulmonary Embolism is present; B) Pulmonary Embolism is not present.”

This prompt format was chosen to align with examination-like reasoning tasks and to reduce LLM refusals common with clinical diagnostic phrasing [6].

Reproducibility and API specifications

All model queries were executed in May 2025. GPT-4o was accessed via the OpenAI API (model: GPT-4o production endpoint). Gemini 2.0 was accessed via the Google Vertex AI API (Gemini 2.0 vision endpoint). Both models were accessed through paid API tiers.

We did not override default provider parameters. Accordingly, GPT-4o calls used provider defaults for temperature, top_p, frequency_penalty, presence_penalty, and max_tokens, while Gemini 2.0 calls used provider defaults for temperature, topP, topK, and maxOutputTokens.

For GPT-4o, each composite grid contained 10 PNG slices in a fixed 5×2 arrangement ordered left to right and top to bottom. For Gemini 2.0, individual PNGs were provided in native slice order.

Bias from prompt framing

We observed that GPT-4o had a higher refusal rate when prompts were more clinically detailed (e.g., when including patient history or requesting the localization of findings). By framing the prompt as a USMLE-style question rather than a real-time clinical case, we reduced refusal frequency and improved answer consistency. Identical diagnostic prompts were applied across both models, although input formats differed (collages for GPT-4o versus single slices for Gemini 2.0). This structure may have also introduced response biases, potentially affecting confidence thresholds or interpretation strategies.

Performance evaluation

**Figure 1:**
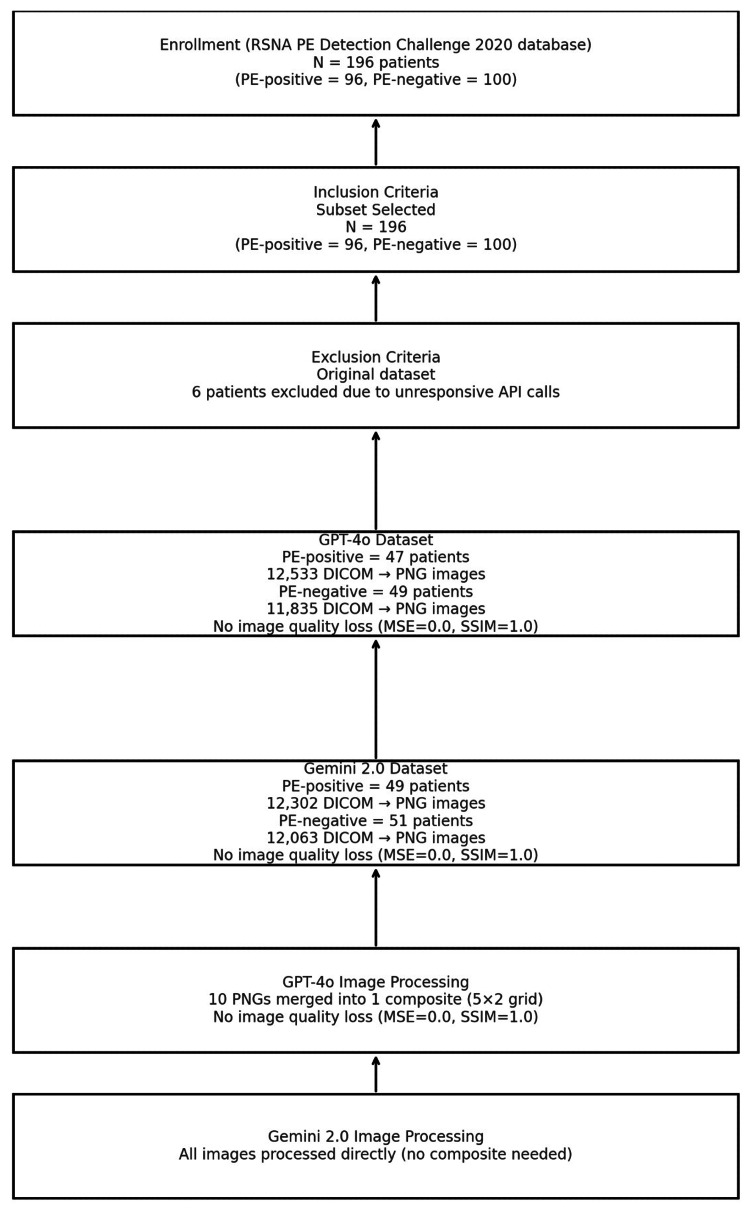
Study enrollment and dataset processing flowchart Flowchart outlining patient enrollment, inclusion/exclusion criteria, dataset creation, and image processing pipelines for GPT-4o and Gemini 2.0. A total of 196 patients from the RSNA PE Detection Challenge 2020 were included, with six excluded due to unresponsive API calls. DICOM images were converted to PNG format for both models, with image fidelity confirmed (MSE=0.0; SSIM=1.0). GPT-4o required composite image batching, while Gemini 2.0 processed full image sets directly RSNA, Radiological Society of North America; PE, pulmonary embolism; API, application programming interface; GPT-4o, GPT-4 Omni; DICOM, Digital Imaging and Communications in Medicine; PNG, Portable Network Graphics; MSE, mean squared error; SSIM, structural similarity index measure

Model outputs were analyzed using standard classification metrics, including accuracy, precision, recall, F1 score, and support. Metrics were calculated separately for PE-positive and PE-negative cases, enabling a granular comparison of each model’s strengths and weaknesses in binary classification. All metrics were reported at the patient level. Figure 1 summarizes the intent to enroll and the image allocation per group across models.

## Results

GPT-4o diagnostic performance

**Figure 2:**
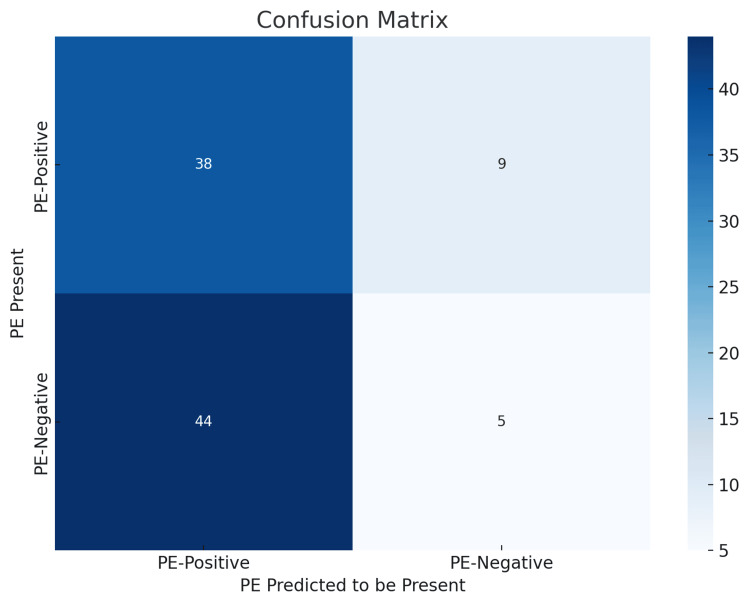
GPT-4o confusion matrix for pulmonary embolism classification Confusion matrix illustrating GPT-4o’s performance in classifying pulmonary embolism (PE) status on CTPA images. The model correctly identified 38 of 47 PE-positive cases and five of 49 PE-negative cases. High false-positive rates reflect a bias toward PE-positive predictions, with strong sensitivity (recall=0.81) but limited specificity (recall=0.10) GPT-4o, GPT-4 Omni; CTPA, computed tomography pulmonary angiography

GPT-4o demonstrated variable performance in classifying CTPA images as either PE-positive or PE-negative. As shown in Figure 2, GPT-4o correctly identified 38 of 47 PE-positive cases (80.9%) but only five of 49 PE-negative cases (10.2%), resulting in a high false-positive rate. This pattern reflects a marked bias toward predicting the presence of PE. GPT-4o’s recall for PE-positive cases was high (0.81), but its precision was moderate (0.46), indicating strong sensitivity but poor specificity. In contrast, the recall for PE-negative cases was low (0.10), and precision was lower (0.36), further highlighting the imbalance in classification performance.

The F1 score for PE-positive predictions was 0.59, compared to just 0.16 for PE-negative predictions, suggesting that while GPT-4o was moderately successful in detecting PE, it performed poorly when ruling out the condition. These diagnostic performance metrics are summarized in Table 2.

**Table 2:** Performance metrics of GPT-4o in classifying pulmonary embolism on CTPA Performance evaluation of GPT-4o using precision, recall, F1 score, and support values for both PE-positive (A) and PE-negative (B) classifications. Metrics were calculated from the model’s predictions on a test set of 96 patients PE, pulmonary embolism; CTPA, computed tomography pulmonary angiography; GPT-4o, GPT-4 Omni; Avg, average

Metric	Precision	Recall	F1 Score	Support
Pulmonary Embolism (A)	0.46	0.81	0.59	47
No Pulmonary Embolism (B)	0.36	0.10	0.16	49
Accuracy	-	-	0.45	96
Macro Avg	0.41	0.46	0.37	96
Weighted Avg	0.41	0.45	0.37	96

Gemini 2.0 diagnostic performance

**Figure 3:**
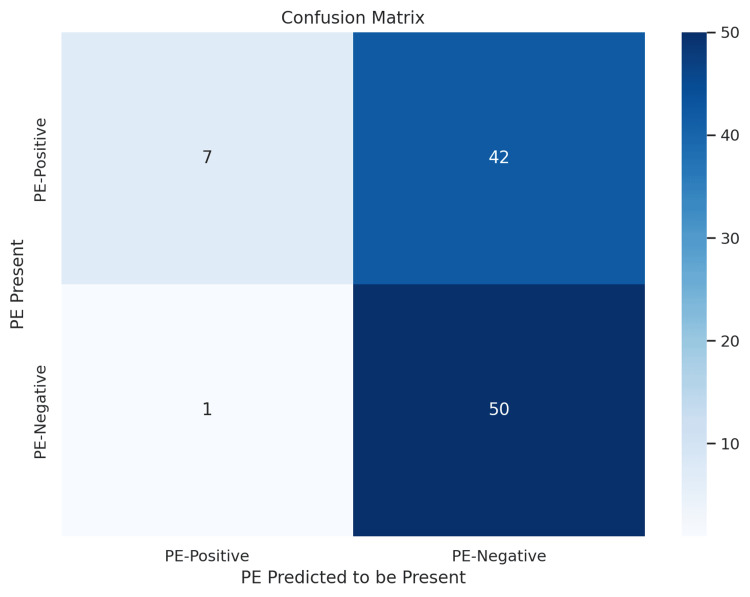
Confusion matrix for Gemini 2.0 model predictions on CTPA images Confusion matrix displaying Gemini 2.0 model performance in identifying pulmonary embolism (PE) from CTPA images. Rows represent the actual presence or absence of PE, while columns indicate the model’s predicted classification. The model correctly identified 50 of 51 PE-negative cases and seven of 49 PE-positive cases. This matrix demonstrates Gemini 2.0’s strong performance in ruling out PE but lower sensitivity in detecting PE presence CTPA: computed tomography pulmonary angiography

Gemini 2.0 showed the opposite trend. As presented in Figure 3, it correctly identified 50 of 51 PE-negative cases (98.0%) but only seven of 49 PE-positive cases (14.3%). This resulted in a high false-negative rate, indicating a strong bias toward classifying patients as PE-negative. Gemini 2.0 had high precision for PE-positive predictions (0.88) but extremely low recall (0.14), suggesting that when it identified PE, it was often correct, but it rarely did so. For PE-negative predictions, recall was excellent (0.98), and precision was moderate (0.54).

F1 scores further supported this trend, with PE-negative predictions scoring 0.70 versus only 0.25 for PE-positive predictions. Overall classification accuracy for Gemini 2.0 was 57%. Detailed performance metrics are shown in Table 3.

**Table 3:** Performance metrics of Gemini 2.0 in detecting pulmonary embolism (PE) from CTPA images Performance evaluation of Gemini 2.0 on the binary classification of PE-positive and PE-negative cases from the RSNA PE Detection dataset. Metrics include precision, recall, F1 score, and support (number of samples) for each class. “Macro Avg” is the unweighted average across classes, while “Weighted Avg” accounts for class imbalance CTPA, computed tomography pulmonary angiography; RSNA, Radiological Society of North America; Avg, average

Metric	Precision	Recall	F1 Score	Support
Pulmonary Embolism (A)	0.88	0.14	0.25	49
No Pulmonary Embolism (B)	0.54	0.98	0.70	51
Accuracy	-	-	0.57	100
Macro Avg	0.71	0.56	0.47	100
Weighted Avg	0.71	0.57	0.48	100

Model comparison and classification bias

**Figure 4:**
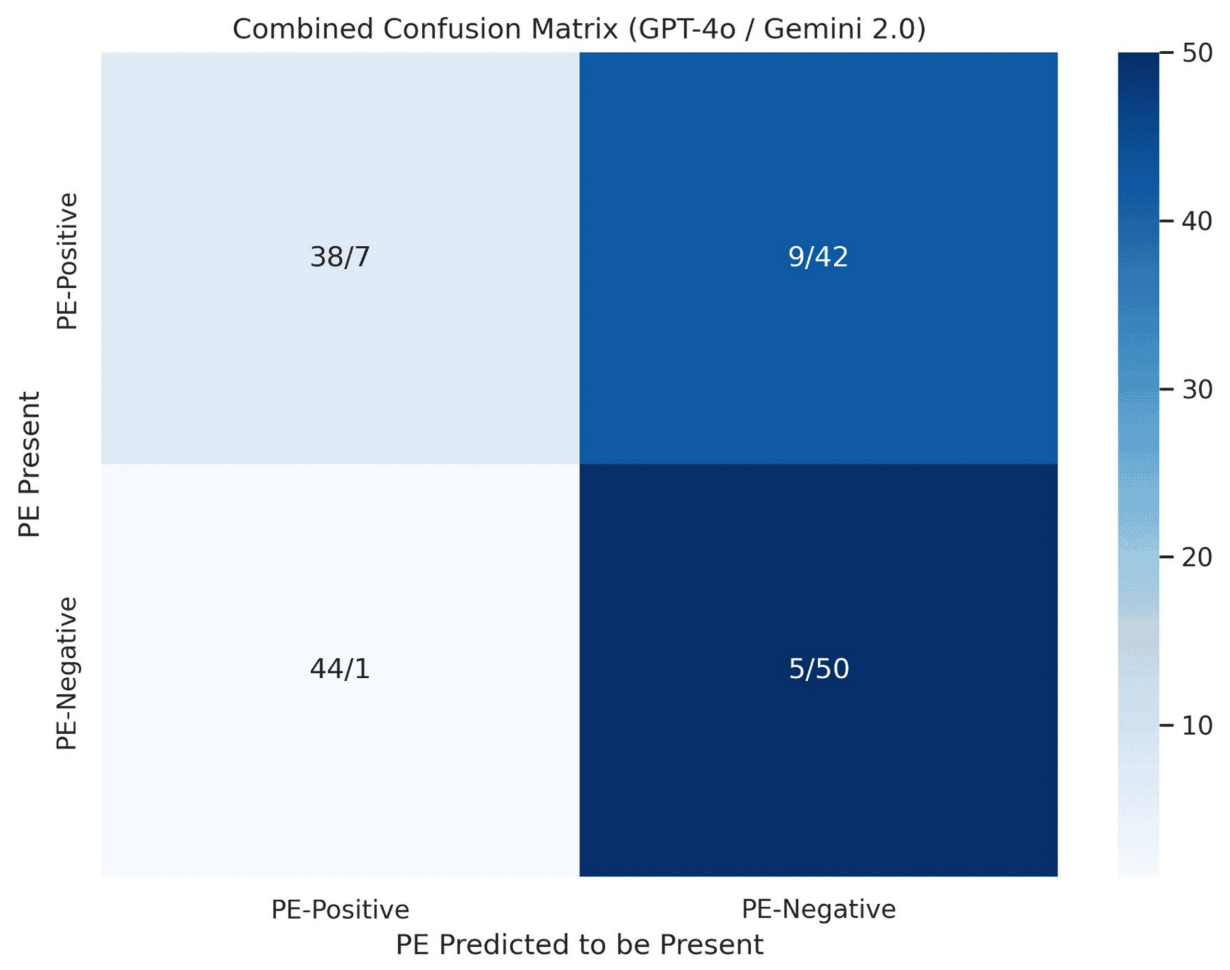
Combined confusion matrix comparing GPT-4o with Gemini 2.0 model predictions Combined confusion matrix illustrating side-by-side classification results for GPT-4o and Gemini 2.0 on pulmonary embolism (PE) status using CTPA imaging. Each cell shows the number of correct or incorrect predictions made by both models in the format: GPT-4o/Gemini 2.0. Rows represent the actual PE status; columns indicate the predicted status CTPA, computed tomography pulmonary angiography; GPT-4o, GPT-4 Omni

When comparing GPT-4o with Gemini 2.0 directly, a distinct inverse pattern emerged. GPT-4o was biased toward detecting PE (high recall and low precision), while Gemini 2.0 was biased toward ruling out PE (high specificity and low recall for positives). These opposing tendencies were evident in both confusion matrices and F1 scores. To visually compare both models’ predictions side by side, we created a combined confusion matrix (Figure 4) that highlights each model’s performance across the same patient cohort.

Both models demonstrated deficiencies in their ability to reliably classify across both PE-positive and PE-negative cases, with GPT-4o underperforming in specificity and Gemini 2.0 underperforming in sensitivity. The trade-off between recall and precision for each model illustrates the current limitations of LLMs in binary radiological classification tasks.

## Discussion

Our findings highlight the variability and limitations of current LLMs when applied to radiological image interpretation. GPT-4o demonstrated high recall for PE-positive cases, suggesting that it may have utility as a screening tool to minimize missed diagnoses. However, its low specificity makes it unsuitable for clinical workflows without radiologist oversight, as false-positive results could lead to unnecessary testing, interventions, and patient anxiety. Conversely, Gemini 2.0 excelled at identifying PE-negative cases, potentially reducing overdiagnosis, but its low recall for PE-positive cases renders it unsafe in scenarios where missing a diagnosis could be fatal. These complementary but inverse biases underscore that neither model is currently fit for independent use in diagnostic medicine.

Comparison with existing AI approaches

While few studies have assessed LLMs directly for radiological interpretation, deep learning convolutional neural network (CNN) models trained on CTPA images have shown markedly higher performance. One study that developed a CNN-long short-term memory (LSTM) hybrid model achieved a sensitivity of 86.6% and a specificity of 93.5%, substantially outperforming both GPT-4o and Gemini 2.0 in balanced diagnostic accuracy [7]. Beyond single architectures, recent advances in meta-learning suggest that models can be adapted to low-data or imbalanced datasets, improving generalizability in medical imaging tasks [8,9]. These approaches highlight that performance gains often depend on specialized training strategies rather than broad multimodal pretraining.

Systematic reviews of LLMs in radiology further reinforce these limitations. A recent synthesis found that ChatGPT and related models show promise in natural language understanding but suffer from variability in accuracy, a lack of transparency, and limited validation on clinical imaging tasks [10]. By contrast, landmark CNN benchmarks such as CheXNet demonstrated radiologist-level performance on pneumonia detection from chest radiographs, achieving superior F1 scores compared with practicing radiologists [11]. Although CheXNet addressed pneumonia rather than pulmonary embolism, its results illustrate the potential of task-optimized CNNs to outperform general-purpose LLMs when applied to image interpretation. This contrast emphasizes that domain-specific optimization remains a key determinant of model performance in radiology.

Limitations of current LLM prompting strategies

Our use of examination-style prompts successfully reduced refusal rates and standardized responses but may have constrained model reasoning strategies. Prompt framing likely induced biases: GPT-4o defaulted to positive classifications, while Gemini 2.0 defaulted to negatives. These findings underscore the importance of developing standardized, clinically validated prompting frameworks if LLMs are to be integrated into radiology.

Clinical implications

Given the high morbidity and mortality of untreated pulmonary embolism, diagnostic systems must demonstrate both high sensitivity (≥90%) and specificity (≥90%) before clinical adoption [2-4]. The current performance of GPT-4o and Gemini 2.0 falls well short of these clinical thresholds. The observed complementarity raises the possibility that ensemble approaches, combining LLMs with CNN-based models or hybrid architectures, could mitigate individual weaknesses. While adjunct or screening roles may eventually be feasible, these applications remain speculative and were not directly tested here. LLMs may provide near-term value in medical education, quality assurance, or as “second readers” that flag potential abnormalities for radiologist review rather than replacing clinical judgment. Finally, the observed negative bias of Gemini 2.0, its tendency to classify patients as PE-negative despite positive findings, requires explicit caution, as this failure mode could be clinically dangerous if unrecognized.

Limitations of this study

This work has several limitations. First, our evaluation was restricted to a single publicly available dataset, which may not fully represent the diversity of real-world CTPA scans across institutions. Second, although we applied identical diagnostic prompts to both models, GPT-4o required composite collages, while Gemini 2.0 analyzed individual slices. This difference in input format may have introduced a subtle source of bias. We attempted to minimize this by keeping prompt framing identical, but further studies should directly compare input formatting effects. Third, both models were tested using their paid API versions (GPT-4o via OpenAI API and Gemini 2.0 via Google Vertex AI), which may differ from free-tier or consumer-facing variants. Fourth, reproducibility remains constrained by API versioning, which evolves rapidly. Finally, both models occasionally produced confident but incorrect predictions (“hallucinations”), underscoring the risk of misclassification if used clinically.

Future directions

Beyond unimodal image interpretation, multimodal frameworks that combine imaging with complementary data sources, such as electronic health records, are increasingly being explored to enhance diagnostic accuracy and support more holistic decision-making [12]. At the same time, evaluations of LLMs in radiology natural language processing pipelines illustrate ongoing challenges: outputs are highly sensitive to prompt phrasing and model bias, reinforcing the need for careful task-specific validation [13].

Research into structured prompting, calibration techniques, and interpretability tools will be essential to reduce variability and build clinician trust. As regulatory bodies such as the FDA begin to define guidance for AI-enabled diagnostics and as radiology societies emphasize integration strategies, systematic benchmarking against gold standard radiologist interpretation will remain critical before widespread adoption [14,15].

## Conclusions

Our comparative evaluation of GPT-4o and Gemini 2.0 highlights both the promise and current limitations of large language models in the diagnostic interpretation of CTPA scans for pulmonary embolism. While GPT-4o demonstrated high recall for PE-positive cases, it struggled with specificity. Conversely, Gemini 2.0 exhibited strong specificity but low sensitivity. These inverse performance trends underscore the variability and bias that can emerge depending on model architecture and prompting strategy.

Until substantial improvements are made in model training, data standardization, and evaluation frameworks, LLMs should not be relied upon as autonomous or standalone diagnostic tools in radiology. Instead, they should be regarded as proof-of-concept adjuncts whose outputs require oversight by trained medical professionals. Future work should focus on optimizing diagnostic prompts, incorporating multimodal training datasets, and validating LLM performance across diverse imaging tasks before clinical integration can be safely considered.
